# Supplementary material for: Quantifying the relationship between SARS-CoV-2 viral load and infectiousness
Source: eLife. 2021 Sep 27;10:e69302. doi: 10.7554/eLife.69302 (PMC8476126; doi:10.7554/eLife.69302)
Supplement: Supplementary file 1. [file elife-69302-supp1.docx]

|  | Parameter estimates (RSE %) | | | | | | | |
| --- | --- | --- | --- | --- | --- | --- | --- | --- |
|  | Models with a fixed incubation period | | | | | | | |
|  | Incubation period = 4 days | | Incubation period = 5 days | | Incubation period = 6 days | | Incubation period = 7 days | |
|  | Fixed effect | Random effect SD | Fixed effect | Random effect SD | Fixed effect | Random effect SD | Fixed effect | Random effect SD |
| $R_{0}$ | 28 (34) | 0.185 (71) | $16.2 (17)$ | $0.23 (48)$ | 23.1 (35) | 0.231 (61) | 23 (29) | 0.17 (97) |
| $\delta(d^{-1})$ | 0.86 (3) | 0.0254 (55) | $0.83 (7)$ | $0.045(72)$ | 0.85 (4) | 0.034 (29) | 0.86 (4.8) | 0.0329 (62) |
| $p$  ($cells^{-1}.d^{-1})$ | $5.4\times{10}^{5}$ (43) | 2.7 (7) | $4.1\times{10}^{5}$  $(77)$ | $2.61 (8)$ | $2.1\times{10}^{6}$ (87) | 2.67 (8.02) | $5.2\times{10}^{6}$ (54) | 2.68 (7.8) |
| $\gamma_{1}$ | 0.38 (28) | 0.94 (40) | 0.38 (20) | 0.98 (28) | 0.38 (19) | 0.86 (30) | 0.37 (20) | 0.87 (30) |
| $\gamma_{2}$ | 0.16 (61) |  | 0.16 (43) |  | 0.17 (41) |  | 0.16 (42) |  |

Supplementary Table 1. Parameter estimates of models with a fixed incubation period ranging from 4 to 7 days.
