# Supplementary material for: Quantifying the relationship between SARS-CoV-2 viral load and infectiousness
Source: eLife. 2021 Sep 27;10:e69302. doi: 10.7554/eLife.69302 (PMC8476126; doi:10.7554/eLife.69302)
Supplement: Supplementary file 2. [file elife-69302-supp2.docx]

|  | Parameter estimates (RSE %) | | | | | | | | | | |
| --- | --- | --- | --- | --- | --- | --- | --- | --- | --- | --- | --- |
|  | Models with different threshold | | | | | | | | | | |
|  | Threshold = 4 log_10_ | | Threshold = 5 log_10_ | | Threshold = 6 log_10_ | | Threshold = 7 log_10_ | | Threshold = 8 log_10_ | |  |
|  | Fixed effect | Random effect SD | Fixed effect | Random effect SD | Fixed effect | Random effect SD | Fixed effect | Random effect SD | Fixed effect | Random effect SD |  |
| $R_{0}$ | 13.9 (17) | 0.34 (26) | 13 (28) | 0.37 (37) | 13.6 (15) | 0.38 (21) | 14.5 (30) | 0.41 (37) | 14.8 (17) | 0.46 (22) |  |
| $\delta$  $(d^{-1})$ | 0.834 (1) | 0.02 (80) | 0.84 (7) | 0.03 (200) | 0.842 (4) | 0.037 (77) | 0.836 (3) | 0.033 (253) | 0.86 (7) | 0.047 (59) |  |
| $p$  ($cells^{-1}.d^{-1})$ | $2.75\times{10}^{5}$  (43) | 2.41 (8) | $2.6\times{10}^{5}$  (44) | 2.4 (9) | $2.8\times10^{5}$  (49) | 2.35 (8) | $3.01\times{10}^{5}$  (78) | 2.3 (9) | $3.8\times{10}^{5}$  (76) | 2.2 (10) |  |
| $\gamma_{1}$ | 0.29 (21) | 0.8 (37) | 0.37 (21) | 0.8 (30) | 0.49 (20) | 0.85 (32) | 0.68 (23) | 0.98 (54) | 1.16 (40) | 1.15 (35) |  |
| $\gamma_{2}$ | 0.13 (46) |  | 0.16 (39) |  | 0.20 (44) |  | 0.25 (63) |  | 0.34 (66) |  |  |
| BIC | 2495 | | 2496 | | 2498 | | 2501 | | 2510 | |  |

Supplementary Table 2. Parameter estimates of models with different threshold values below which the transmission is set to 5%.
