# Supplementary material for: Quantifying the relationship between SARS-CoV-2 viral load and infectiousness
Source: eLife. 2021 Sep 27;10:e69302. doi: 10.7554/eLife.69302 (PMC8476126; doi:10.7554/eLife.69302)
Supplement: Supplementary file 3. [file elife-69302-supp3.docx]

Supplementary Table 3: Parameters estimates of the Model M2 (left) and the same model without variability in transmission (Right).

|  | Parameter estimates (RSE%) | | | |
| --- | --- | --- | --- | --- |
|  | Logit-Linear (M2) | | Model without variability | |
|  | Fixed effect | Random effect SD | Fixed effect | Random effect SD |
| $R_{0}$ | $13.6 (15)$ | $0.38 (21)$ | 11 (18) | 0.376 (24) |
| $\delta(d^{-1})$ | $0.84 (4)$ | $0.037 (77)$ | 0.87 (1.7) | 0.0342 (32.4) |
| $p$  ($cells^{-1}.d^{-1})$ | $2.8\times{10}^{5} (50)$ | $2.35 (8)$ | $2\times10^{5}$ (54) | 2.45 (8.28) |
| $\gamma_{1}$ | 0.49 (20) | $0.85 (32)$ | 0.712 (13.6) | $-$ |
| $\gamma_{2}$ | 0.21 (44) |  | 0.39 (22.4) |  |
| -2 x log-likelihood | 2432 | | 2443 | |
