# Supplementary material for: Quantifying the relationship between SARS-CoV-2 viral load and infectiousness
Source: eLife. 2021 Sep 27;10:e69302. doi: 10.7554/eLife.69302 (PMC8476126; doi:10.7554/eLife.69302)
Supplement: Supplementary file 4. — Secondary attack rate calculated for each category (Right). [file elife-69302-supp4.docx]

| Proportion of contacts | | | | | Secondary attack rate | | | |
| --- | --- | --- | --- | --- | --- | --- | --- | --- |
| Mask use | | | | | Mask use | | | |
|  | Never | Always | Unknown | Total | Never | Always | Unknown | Total |
| Non-Household | 11/582  2 % | 146/582  25 % | 76/582  13 % | 233/582  40 % | 2/11  18 % | 18/146  12 % | 9/76  12 % | 29/233  12 % |
| Household | 129/582  22 % | 93/582  16 % | 127/582  22 % | 349/582  60 % | 23 / 129  18 % | 25/93  27 % | 39/127  31 % | 87/349  25 % |
| Total | 140/582  24 % | 239/582  41 % | 203/582  35% |  | 25/140  18 % | 43/239  18 % | 48/203  24 % |  |

Supplementary file 4: Proportion of contacts wearing masks in each category (left). Secondary attack rate calculated for each category (right).
