# Supplementary material for: Quantifying the relationship between SARS-CoV-2 viral load and infectiousness
Source: eLife. 2021 Sep 27;10:e69302. doi: 10.7554/eLife.69302 (PMC8476126; doi:10.7554/eLife.69302)
Supplement: Transparent reporting form [file elife-69302-transrepform.docx]

***eLife’s* transparent reporting form**

We encourage authors to provide detailed information *within their submission* to facilitate the interpretation and replication of experiments. Authors can upload supporting documentation to indicate the use of appropriate reporting guidelines for health-related research (see [EQUATOR Network](about:blank)), life science research (see the BioSharing Information Resource), or the [ARRIVE guidelines](about:blank) for reporting work involving animal research. Where applicable, authors should refer to any relevant reporting standards documents in this form.

If you have any questions, please consult our Journal Policies and/or contact us: [editorial@elifesciences.org](about:blank).

**Sample-size estimation**

- You should state whether an appropriate sample size was computed when the study was being designed
- You should state the statistical method of sample size computation and any required assumptions
- If no explicit power analysis was used, you should describe how you decided what sample (replicate) size (number) to use

Please outline where this information can be found within the submission (e.g., sections or figure legends), or explain why this information doesn’t apply to your submission:

The data used in this study originate from an original clinical study. Details on study can be found Marks M. *et al.* Transmission of COVID-19 in 282 clusters in Catalonia, Spain: a cohort study .

Consequently no sample size was performed for this ad hoc modelling study.

**Replicates**

- You should report how often each experiment was performed
- You should include a definition of biological versus technical replication
- The data obtained should be provided and sufficient information should be provided to indicate the number of independent biological and/or technical replicates
- If you encountered any outliers, you should describe how these were handled
- Criteria for exclusion/inclusion of data should be clearly stated
- High-throughput sequence data should be uploaded before submission, with a private link for reviewers provided (these are available from both GEO and ArrayExpress)

Please outline where this information can be found within the submission (e.g., sections or figure legends), or explain why this information doesn’t apply to your submission:

The data come from a clinical study, hence no replicates have been performed.

Some outliers were removed from the original dataset. The selection process is described in the section *Study participant* (page 12) in the methods and is summarized in the supplementary figure S1 (page 19).

No High-throughput techniques was used in this study.

**Statistical reporting**

- Statistical analysis methods should be described and justified
- Raw data should be presented in figures whenever informative to do so (typically when N per group is less than 10)
- For each experiment, you should identify the statistical tests used, exact values of N, definitions of center, methods of multiple test correction, and dispersion and precision measures (e.g., mean, median, SD, SEM, confidence intervals; and, for the major substantive results, a measure of effect size (e.g., Pearson's r, Cohen's d)
- Report exact p-values wherever possible alongside the summary statistics and 95% confidence intervals. These should be reported for all key questions and not only when the p-value is less than 0.05.

Please outline where this information can be found within the submission (e.g., sections or figure legends), or explain why this information doesn’t apply to your submission:

We made use of non-linear mixed effect modelling in order to estimate the parameters of the different models. We computed the maximum-likelihood estimator using the stochastic approximation expectation-maximization (SAEM) algorithm implemented in Monolix Software 2020R1. The BIC was used to select the model providing the best fit.

The association between the viral load and the transmission probability was assessed using a Wald test. All statiscial methods are detailed in the sections *statistical model for viral kinetics, probability of transmission* and *parameters estimation* in the methods (details pages 15 to 17).

Raw data are given as follows:

- Viral load and sampling times in index cases are plotted in figure 1 for individual having 3 swab samples
- The distribution of contacts is depicted in the supplementary figure 2

(For large datasets, or papers with a very large number of statistical tests, you may upload a single table file with tests, Ns, etc., with reference to sections in the manuscript.)

**Group allocation**

- Indicate how samples were allocated into experimental groups (in the case of clinical studies, please specify allocation to treatment method); if randomization was used, please also state if restricted randomization was applied
- Indicate if masking was used during group allocation, data collection and/or data analysis

Please outline where this information can be found within the submission (e.g., sections or figure legends), or explain why this information doesn’t apply to your submission:

As we realised a posterior analysis of previously published data, no groups were allocated.

The details of the original study can be found in: Marks M. *et al.* Transmission of COVID-19 in 282 clusters in Catalonia, Spain: a cohort study.

**Additional data files (“source data”)**

- We encourage you to upload relevant additional data files, such as numerical data that are represented as a graph in a figure, or as a summary table
- Where provided, these should be in the most useful format, and they can be uploaded as “Source data” files linked to a main figure or table
- Include model definition files including the full list of parameters used
- Include code used for data analysis (e.g., R, MatLab)
- Avoid stating that data files are “available upon request”

Please indicate the figures or tables for which source data files have been provided:

The dataset used for this study has already been uploaded.

The model files and the simulations codes will be available on FigShare upon the paper acceptance. This will ensure appropriate referencing of data and source code used.
